# Life-threatening paradoxical thromboembolism in a patient with patent foramen ovale

**DOI:** 10.1186/s12947-022-00298-x

**Published:** 2022-11-28

**Authors:** Antonio Rizza, Francesco Negro, Tommaso Gasbarri, Roberto Arpesani, Baldassare Ferro, Paolo Roncucci, Cataldo Palmieri, Simone Sorbo, Emilio Maria Pasanisi, Marco Solinas, Sergio Berti

**Affiliations:** 1 Cardiology Unit, Ospedale del Cuore, Fondazione Toscana “G. Monasterio”, Massa, Italy; 2 Cardiology Division, Pisa University Hospital, Via Paradisa 2, Pisa, Italy; 3 Cardiac Surgery Department, Fondazione Tosca “G. Monasterio”, Ospedale del Cuore, Massa, Italy; 4 Interventional Radiology Unit, Spedali Riuniti Di Livorno, Livorno, Italy; 5 Intensive Care Unit, Spedali Riuniti Di Livorno, Livorno, Italy; 6 Cardiology Unit, Spedali Riuniti Di Livorno, Livorno, Italy

**Keywords:** Pulmonary embolism, Deep vein thrombosis, Patent foramen ovale, Paradoxical embolization

## Abstract

**Background:**

Venous thromboembolism represents the third most frequent acute cardiovascular syndrome worldwide. Its clinical manifestations are deep vein thrombosis and/or pulmonary embolism. Despite a considerable mortality, diagnosis is often missed.

**Case presentation:**

We report the management of a female patient with high-risk pulmonary thromboembolism treated initially with thromboaspiration, complicated by embolus jailing in a patent foramen ovale. In this situation, left cardiac chambers and systemic circulation were jeopardized by this floating embolus.

**Conclusions:**

High-risk pulmonary embolism requires reperfusion strategy but sometimes mechanical thromboaspiration may be not fully successful; transesophageal echocardiography led to a prompt diagnosis of this unexpected finding; in this very particular case, open surgery represented a bail-out procedure to avoid cerebral and systemic embolism.

**Supplementary Information:**

The online version contains supplementary material available at 10.1186/s12947-022-00298-x.

## Background

Pulmonary embolism (PE) leads to variable symptoms, making the diagnosis challenging. Both American and European guidelines classify patients presenting with cardiac arrest or hemodynamic instability at high risk of mortality [1, 2]. Notably, in American guidelines, subjects with syncope or “*thrombus in transit*” at imaging are also considered at high risk. In this setting, reperfusion therapy is mandatory. This can be achieved by systemic thrombolysis or invasive approaches, such as catheter directed therapy or surgical embolectomy.

Patent foramen ovale (PFO) is defined as a small communication between right and left atrium at level of fossa ovale. This condition can be found in almost 25% of population, thus its correlation with cryptogenic stroke is still debated and no clear recommendations on its management exist [3].

## Case presentation

A 55-year-old female patient was admitted to a peripheral hospital intensive care unit for type 1 respiratory failure complicated by shock and lactic acidosis (heart rate 106 bpm, blood pressure 60/30 mmHg, respiratory rate 25 breaths per minute and oxygen saturation 90% in air). No clinical signs of deep vein thrombosis (DVT) were observed. Blood exams revealed normal hemoglobin and red blood cell count, 12.000 white blood cells/µl, creatinine 1.8 mg/dl, NT-proBNP 19,800 pg/ml, D-dimer > 8000 ng/ml and lactates 8.6 mEq/l.

The patient had severe obesity [body mass index (BMI) 47 kg/m2] and no other cardiovascular risk factors. She complained effort-induced dyspnea in the last 2 days, and she was not on medications. The patient had an accidental fall with consequent cranial trauma in the previous month.

**Fig. 1:**
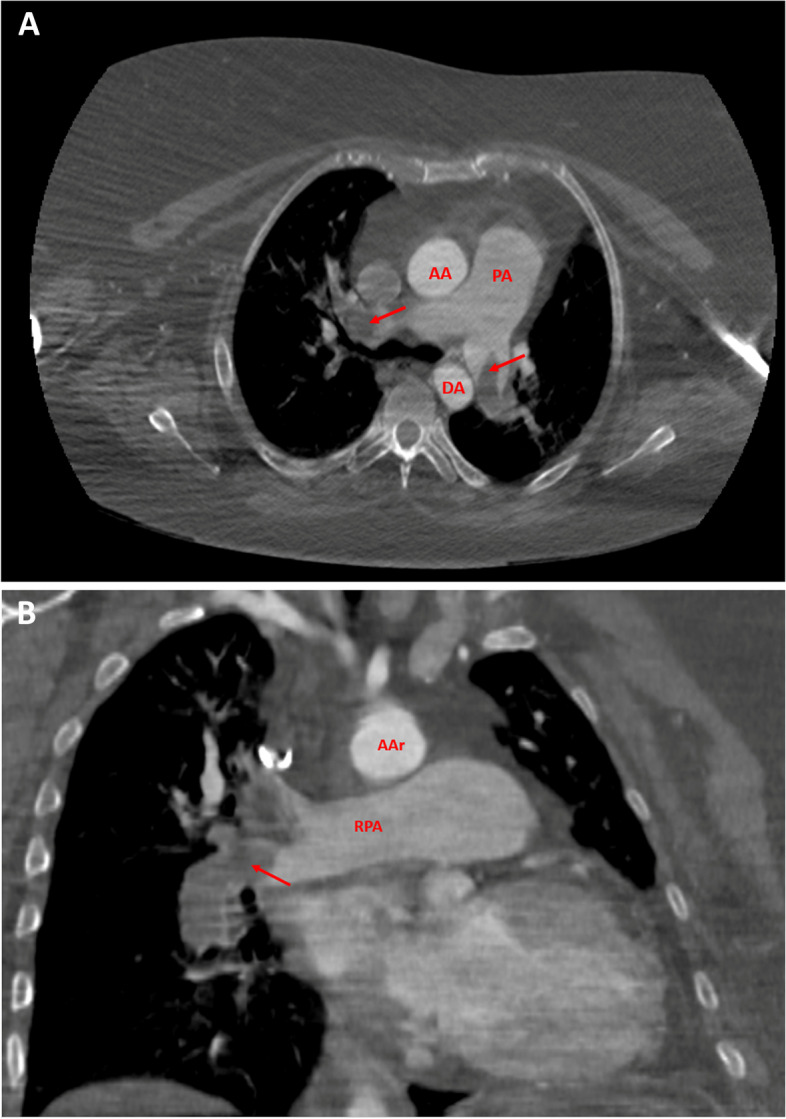
**A-B** Computed tomography pulmonary angiogram in axial scan (**A**) coronal scan (**B**). Bilateral pulmonary artery emboli are shown by red arrows. AA = ascending aorta. AAr = aortic arc.; DA = descending aorta. PA = pulmonary artery. RPA = right pulmonary artery

Urgent echocardiogram showed severe right ventricular systolic dysfunction and elevated systolic pulmonary artery pressure (80 mmHg). Unfractionated heparin infusion was started, and computed tomography pulmonary angiogram showed massive bilateral pulmonary embolism (Fig. 1A-B).

**Fig. 2:**
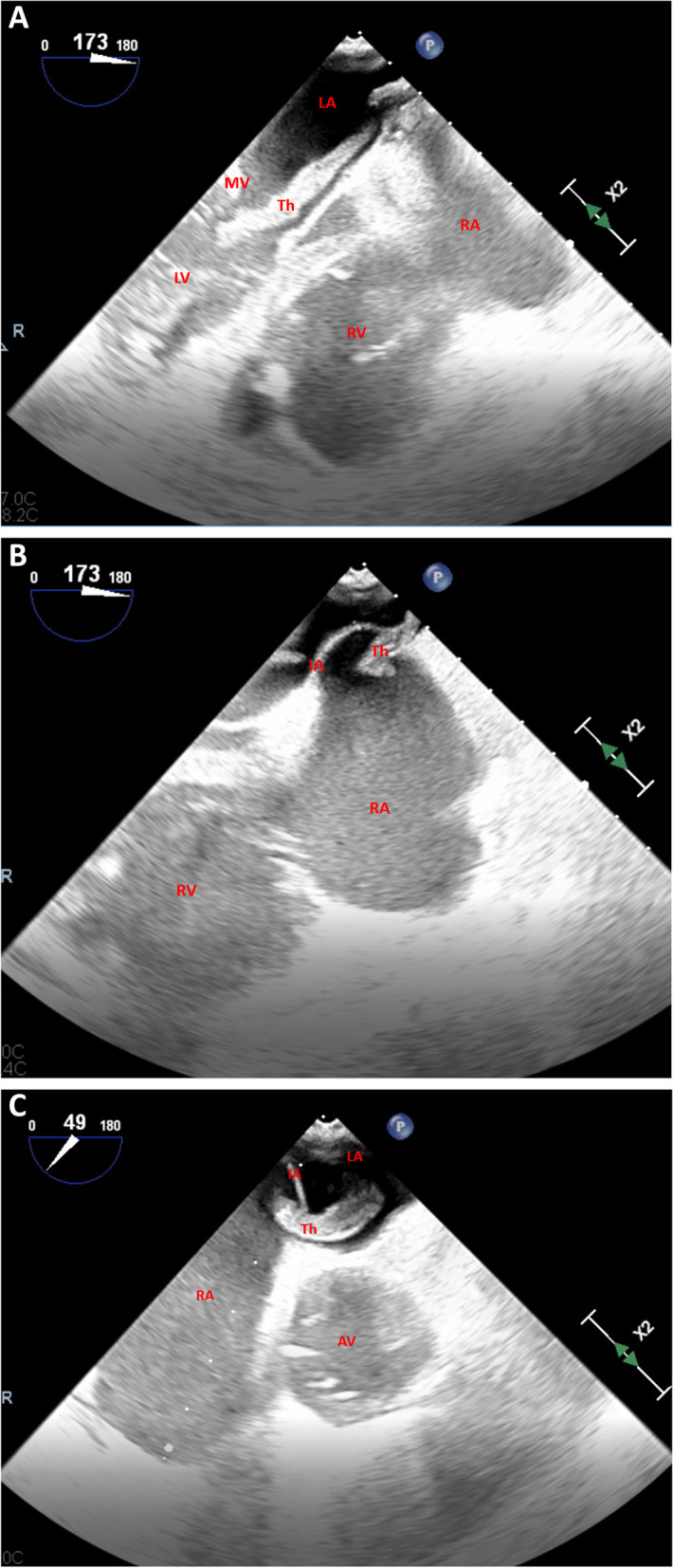
**A-C** Transesophageal echocardiogram; inverted four chamber midesophageal scan (**A-B**) shows dilated right chambers and a thrombus involving left atrium, right atrium and mitral valve; short axis midesophageal scan (**C**) shows thrombus crossing the interatrial septum. AV = aortic valve. IA = interatrial septum. LA = left atrium. MV = mitral valve. RA = right atrium. Th = thrombus

**Fig. 3:**
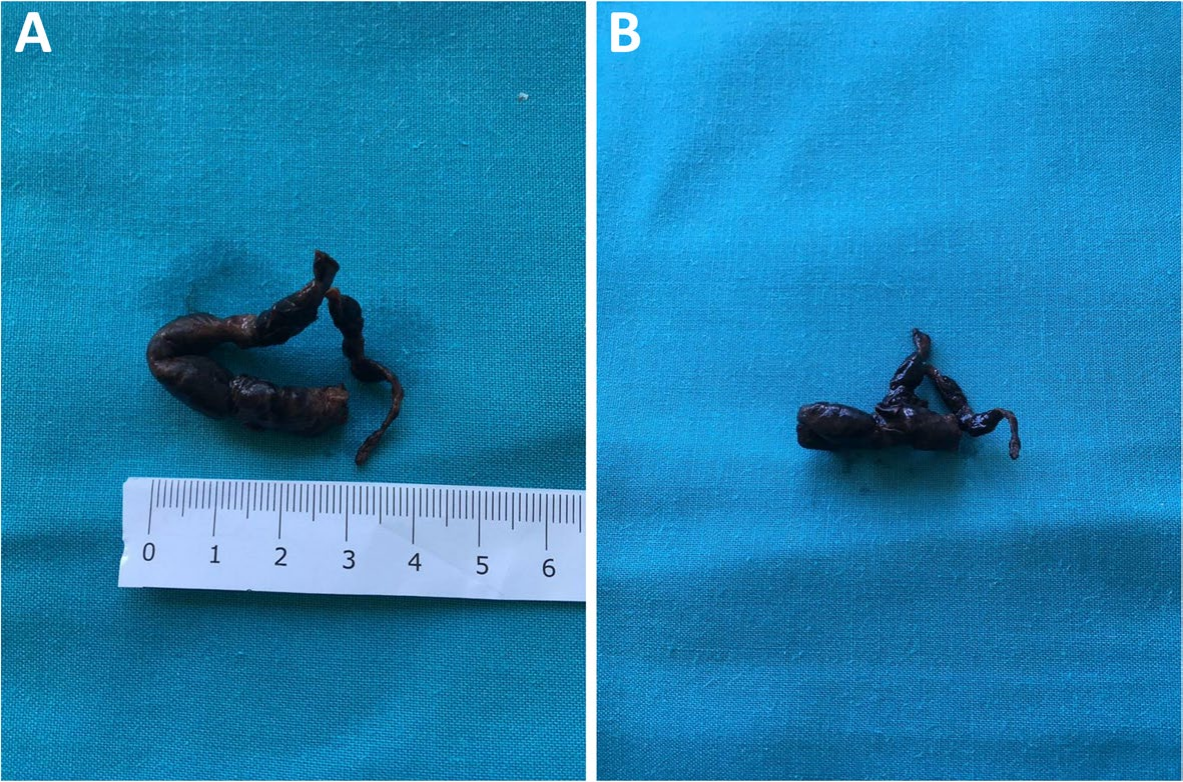
**A-B** Real images of thrombus preserved in formalin after surgical intervention

Urgent reperfusion strategy was mandatory due to hemodynamic instability, but systemic thrombolytic therapy was rejected because of recent head trauma; therefore, percutaneous thromboaspiration was performed. Although respiratory and hemodynamic parameters improved after the procedure, a transesophageal echocardiogram showed a new hyperechogenic mass in atrial septum, floating in both left and right atria and involving mitral valve (Fig. 2A-C, Additional file 1: Video 1, Additional file 2: Video 2, Additional file 3: Video 3, Additional file 4: Video 4 and Additional file 5: Video 5). The patient was immediately transferred to “Ospedale del Cuore” cardiac surgery department to remove the mass and avoid paradoxical embolism. During the intervention, a worm-shaped thrombus (10 × 1 cm) jailed in a 2 cm wide PFO was observed (Fig. 3A-B); consequently, thrombus was removed and PFO sutured.

## Discussion and conclusions

In our case, PFO was discovered accidentally because of a large embolus was trapped inside it. This phenomenon could be facilitated by elevated right cardiac chambers pressure and mechanical thromboaspiration, but it is unclear whether to consider it a procedural complication or a new embolic manifestation. In this case, cardiac surgery was pursued due to high systemic and cerebral embolization risk.

Left popliteal vein thrombosis was found at ultrasound performed after the surgical intervention. The patient was weaned from inotropes and vasopressor after 1 week and discharged after 1 month with mild right ventricular systolic dysfunction and moderate pulmonary hypertension. Direct oral anticoagulant and home oxygen therapy was prescribed. She is currently on follow-up for chronic thromboembolic pulmonary hypertension (CTEPH) management.

High-risk pulmonary embolism is a life-threatening condition that must be managed with reperfusion strategy by medical or invasive approaches. Mechanical thromboaspiration could be complicated by incomplete emboli removal. In this case, a residual thrombus jailed in a PFO was successfully removed by a surgical procedure.

## Supplementary Information

**Additional file 1:** **Video 1.** Inverted four chamber midesophageal scan. Anenlarged right ventricle and a floating thrombus between the right atrium andleft atrium can be observed.

**Additional file 2:** **Video 2.** Short axis midesophageal scan. A floatingthrombus, crossing the interatrial septum, can be observed.

**Additional file 3:** **Video 3.** Four chamber midesophageal scan. A thrombusinvolving the mitral valve can be observed.

**Additional file 4:** **Video 4.** Two chamber midesophageal scan. A thrombusinvolving the mitral valve can be observed.

**Additional file 5:** **Video 5.** 3D transesophageal echocardiogram showing thefloating thrombus.

## Data Availability

All data generated or analysed during this study are included in this published article (and its supplementary information files).

## Abbreviations

BMI: Body mass index
CTEPH: Chronic thromboembolic pulmonary hypertension
DVT: Deep vein thrombosis
PAPs: Systolic pulmonary artery pressure
PE: Pulmonary embolism
PFO: Patent foramen ovale
